# Novel Spectrum Sensing Algorithms for OFDM Cognitive Radio Networks

**DOI:** 10.3390/s150613966

**Published:** 2015-06-15

**Authors:** Zhenguo Shi, Zhilu Wu, Zhendong Yin, Qingqing Cheng

**Affiliations:** 1 School of Electronics and Information Engineering, Harbin Institute of Technology, 92 Xidazhi Street, Harbin150001, China; E-Mails: shizhenguotvt@gmail.com (Z.S.); yinzhendong@hit.edu.cn (Z.Y.); cqq@hit.edu.cn (Q.C.); 2 Department of engineering, Faculty of Science and Engineering, Macquarie University, North Ryde, Sydeny, NSW 2109, Australia

**Keywords:** cognitive radio, spectrum sensing, OFDM, CP detection, PT detection

## Abstract

Spectrum sensing technology plays an increasingly important role in cognitive radio networks. Consequently, several spectrum sensing algorithms have been proposed in the literature. In this paper, we present a new spectrum sensing algorithm “Differential Characteristics-Based OFDM (DC-OFDM)” for detecting OFDM signal on account of differential characteristics. We put the primary value on channel gain *θ* around zero to detect the presence of primary user. Furthermore, utilizing the same method of differential operation, we improve two traditional OFDM sensing algorithms (cyclic prefix and pilot tones detecting algorithms), and propose a “Differential Characteristics-Based Cyclic Prefix (DC-CP)” detector and a “Differential Characteristics-Based Pilot Tones (DC-PT)” detector, respectively. DC-CP detector is based on auto-correlation vector to sense the spectrum, while the DC-PT detector takes the frequency-domain cross-correlation of PT as the test statistic to detect the primary user. Moreover, the distributions of the test statistics of the three proposed methods have been derived. Simulation results illustrate that all of the three proposed methods can achieve good performance under low signal to noise ratio (SNR) with the presence of timing delay. Specifically, the DC-OFDM detector gets the best performance among the presented detectors. Moreover, both of the DC-CP and DC-PT detector achieve significant improvements compared with their corresponding original detectors.

## 1. Introduction

Recently, with the rapid development of wireless communication applications, the problem of spectrum scarcity has become more serious than ever before [1,2]. Therefore, how to tackle the conflict between the spectrum scarcity and spectrum utilization has become a dramatically critical issue [3]. Cognitive radio (CR) which has the ability to detect and share the unutilized spectrum has been employed as a promising solution to this problem [4–6]. In cognitive radio networks, one of the most challenging and crucial works is spectrum sensing. In order to avoid interfering with the primary users, the spectrum sensing algorithms should have the capacity to catch the presence of the primary users within a short time especially under very low signal to noise ratio (SNR) regions [7].

A number of spectrum sensing algorithms have been presented and analyzed in the literature such as matched filtering [8], energy detection [9], cyclostationary detection [10] and covariance detection [11], etc. All of them have corresponding merits and demerits. For example, energy detection is one of the most basic sensing methods, which does not need any prior information of the signal. However, it is too sensitive to the noise variance, and the uncertainty in noise variance causes significant degradation of the performance [12]. Although other sensing algorithms may be robust to the noise uncertainty, they have to know the structure of the signal. For instance, matched filter detection needs the waveform of the transmitted signal, and the cyclostationary detection requires the cyclic period of the primary users, respectively. In addition, in [13] the authors offered the locally optimal detector to sense the interested spectrum. This algorithm mainly discussed about detecting the signals modulated by BPSK, which could get better performance than the energy detector. However, the effect of timing delay was not considered in this paper.

Orthogonal frequency division multiplexing (OFDM), which is one of the most popular communication schemes in the current communication system, is a good candidate for cognitive radio data transmission for its capability of combating multi-path fading and mitigating intersymbol interference (ISI) [14–16]. Therefore, it is fair to assume that the spectrum sensing algorithms should be able to sense the existence of primary users under the OFDM scheme. Numbers of previous works on OFDM spectrum sensing have been studied and reported in the literature by using the structure features of OFDM signals [17,18]. In [19], the authors proposed optimal and sub-optimal Neyman-Pearson (NP) spectrum sensing methods to detect the OFDM signals based on the feature of cyclic prefix and also studied the generalized likelihood ratio test (GLRT) according to the second order statistic of the received OFDM signals. However, the presented approaches did not deeply analyze the effect of channel and the optimal algorithm was sensitive to the noise uncertainty. The pilot tones (PT) detection was discussed in [7,20]. The detection performance of these algorithms has been improved, but the computational complexity have increased as the cost. Besides, there have been a number of works studying the CP detector [21,22]. Although the performance of these algorithms were improved, they were limited by the length of CP and decreased with the presence of timing delay.

Some literatures have proposed a number of algorithms to solve these problems presented above. In [15], the authors provided an improved CP detector by constructing a likelihood ratio test (LRT) based on the multivariate p.d.f.'s of a particular autocorrelation vector. The new detector can get an accurate threshold without estimating timing delay. But performance of this detector is a little worse than the optimal CP detector. Moreover, noise uncertainty is an important factor which could impact the performance of the sensing algorithms [23,24]. Thus the spectrum sensing method should have a certain extent robustness of noise uncertainly. Some new spectrum sensing algorithms are discussed in [25], which are robust against noise variance uncertainty. However, the detection performance of these algorithms is not good enough compared with other algorithms. In [17], specific detection algorithms (feature match) are presented. Although these proposed sensing methods are robust to frequency offset and noise power uncertainty, they could not perform perfect spectrum sensing for sensing errors. Therefore, finding an effective sensing algorithm to perform accurate spectrum sensing is an argent work.

In this paper, three novel spectrum sensing algorithms based on derivative features are proposed to detect OFDM signals. The first way is named “Differential Characteristics-Based Orthogonal Frequency Division Multiple (DC-OFDM)” algorithm. It is a suboptimal OFDM signal detector considering the effect of the channel fading and timing delay. The second approach is “Differential Characteristics-Based Cyclic Prefix (DC-CP)” algorithm, which utilizes the property of CP to sense the spectrum. And the auto-correlation vector instead of a single auto-correlation value is taken to catch the desired signals. The third algorithm is “Differential Characteristics-Based Pilot Tones (DC-PT)” algorithm. It takes advantage of frequency-domain cross-correlation based on pilot tones feature. The DC-CP and DC-PT detectors could be seen as special applications of DC-OFDM detector. All of the proposed methods can get the satisfied performance compared with other approaches. More specifically, the contributions of this paper can be summarized as follows.

(1) A DC-OFDM spectrum sensing algorithm which takes the advantage of differential characteristics is presented in this paper. Compared with other detectors, the DC-OFDM detector achieves better detection performance.
(2) Considering the effect of timing delay (*τ*), the expressions for the test statistics of DC-OFDM with different *τ* are proposed (see Equations (17)–(19)). Furthermore, the DC-OFDM detector provides a novel way by utilizing the differential characteristics to sense the OFDM signals.
(3) DC-CP detector is brought forward, which is derived from the traditional CP detector by employing the differential operation. Compared to the traditional one, the performance of the DC-CP detector is obviously better.
(4) Based on the differential characteristics, we propose a new PT detector through frequency-domain cross-correlation, and also derive the test statistic of DC-PT detector.
(5) We derive the theoretical expressions of *P_m_* and *P_f_* of the three proposed detectors, which are based on the probability distribution functions for the different test statistics under *H*_0_ and *H*_1_ From these theoretical results, it is easy to get the corresponding thresholds for any given *P_f_*.
(6) We perform extensive simulations to demonstrate the theoretical results and prove the promising detectors.

The rest of the paper is now organized as follows. The system model of the OFDM signal is described in Section “System Model and Hypothesis Test”. In Section “Algorithm of DC-OFDM Detection” and Section “DC-CP Detection and DC-PT Detection”, three new sensing algorithms based on derivative features are separately discussed. The expressions of *P_f_* and *P_m_* for the proposed detectors are derived in Section “Probabilities of Misdetection and False Alarm”. Then we provide simulation results and compare the performance of our proposed methods with other algorithms in Section “Simulation Results”. Finally, conclusions are given in the last Section “Conclusions”.

## 2. System Model and Hypothesis Test

**Figure 1:**
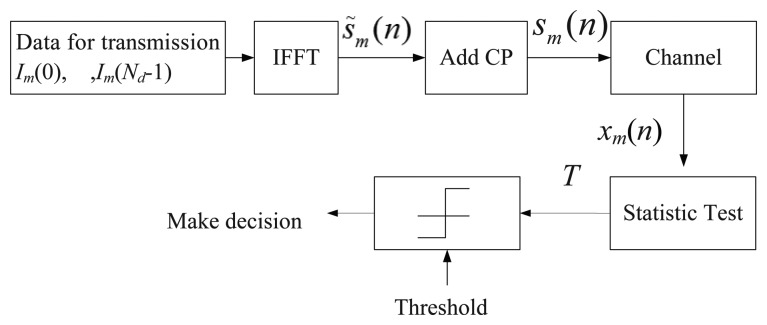
The traditional OFDM spectrum sensing structure in a cognitive radio.

In this section, we will briefly describe the structure of OFDM system at first. After that, we will discuss the issue of hypothesis test. A simplified Block diagram of an OFDM system is shown in Figure 1. For a generic OFDM transmitter, the *m*th block of data symbols *I_m_*(0), …,*I_m_*(*N_d_* − 1) are mapped onto the subcarriers, where *N_d_* is the data block size. And after the inverse fast Fourier transform (IFFT), we will get the time-domain signals *s̃_m_*(0),…,*s̃_m_* (*Nd* − 1), where

(1) $${\tilde{s}}_{m}(n)=\sqrt{\frac{E_{s}}{N_{d}}}\overset{N_{d}/2-1}{\underset{\text{k}=-N_{d}/2}{\text{∑}}}I_{m}(k){\text{e}}^{j\pi nk/N_{d}}$$

**Figure 2:**
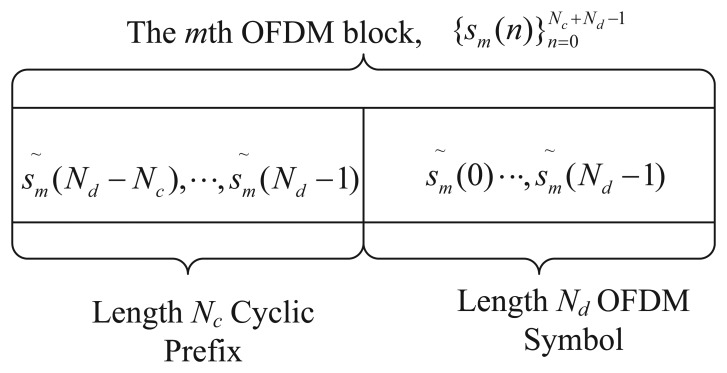
The traditional structure of the transmitted *m*th OFDM block.

*E_s_* denotes the power of each transmitted symbol, and we assume the power is unit (*E_s_* = 1). Without loss of generality, the IFFT block size is taken to be *N_d_*. Figure 2 illustrates the structure of the *m*th block of an OFDM transmission, where *N_c_* is the length of CP As is shown, the CP is added by putting an exact copy of *s̃_m_*(*N_d_* − *N_c_*),…, *s̃_m_*(*N_d_* − 1) at the front of the block. So, we get the transmitted OFDM symbol ${\left\{s_{m}\left(n\right)\right\}}_{n=0}^{N_{c}+N_{d}-1}$.

In practice, the transmitters and receivers are difficult to be synchronous. Therefore, let *τ* be the synchronization mismatch, where 0 ⩽ *τ ≤ N_c_* + *N_d_*. After passing through fading channel, the received signal of the *m*th block can be expressed as

(2) $$x_{m}(\text{n})={\text{hs}}_{m}(\text{n}-\tau )+{\text{e}}_{m}(\text{n}),\text{n}=0,1,\ldots ,N_{c}+N_{d}-1$$

where *m* = 0,…, *M* − 1 and *M* is the total number of received OFDM blocks; *h* is the channel gain; *e_m_*(*n*) is the complex additive white Gaussian noise (AWGN) with zero-mean and variance $\sigma_{n}^{2}$, and the SNR is defined as ${\left|h\right|}^{2}/\sigma_{n}^{2}$ at the receiver.

Consider two binary hypotheses, *H*_1_ and *H*_0_, where *H*_1_ and *H*_0_ denote the presence and absence of a primary user, respectively. For the spectrum sensing, the object is to decide whether the received signal consists of primary user's signal. Consequently, the two binary hypotheses can be written as:

(3) $$\begin{array}{lll} H_{0} & :x_{m}(n) & =e_{m}(n),\\ H_{1} & :x_{m}(n) & =hs_{m}(n-\tau )+e_{m}(n) \end{array}$$

Let *θ* = |*h*|, it can be clearly seen from (3) if *θ* = 0, the received signal is only composed of noise. Otherwise it consists the signal plus noise. So taking interesting of *θ*, Equation (3) can be rewritten as

(4) $$\begin{array}{ccc} H_{0}:\theta =0 & \text{and} & H_{1}:\theta >0 \end{array}$$

## 3. Algorithm of DC-OFDM Detection

In this section, we will propose a new detector (DC-OFDM detector) to sense to OFDM signals. The new detector, based on the NP criterion, utilizes the merits of differential characteristics to sense the interested spectrum.

Let **x** = [*x*_0_(0), *x*_0_(1), …, *x*_0_ ((*N_c_*+*N_d_* − 1),…, *x_M_* (*N_c_* + *N_d_* − 1)]*^t^* is the received vector signal and *M* is the total number of received OFDM blocks. According to the NP criterion, the traditional detection based on the LRT is:

(5) $$T=\log(\frac{p(x|H_{1})}{p(x|H_{0})})\overset{H_{0}}{\underset{H_{1}}{≶}}\gamma$$

where *T* is the test statistic; *p*(.) denotes the probability density function (p.d.f) and *γ* is the threshold. According to Equation (4), detecting the absence and presence of the primary user can be alternated to test *θ* = 0 against *θ* > 0, especially in the case of weak signal detection. Since *θ* around zero is our primary concern, we can make the differential operation of *θ* to get the locally optimal solution when *θ* = 0. Then the test statistic can be written as [26]:

(6) $$T=\frac{{p^{\left(\text{n}\right)}(x|\theta )|}_{\theta =0}}{p(x|0)}$$

where *p*^(^*^n^*^)^(**x**|*θ*) := *d^n^p*(**x**|*θ*)/*dθ^n^*. Under *H*_0_, *θ* = 0, the received signal only contains the noise, so the elements of x are independent. The p.d.f of *p*(**x**) can be expressed as

(7) $$\begin{array}{ll} p(x|H_{0}) & =\overset{M-1}{\underset{m=0}{\text{∏}}}\overset{N_{c}+N_{d}-1}{\underset{\text{i}=0}{\text{∏}}}p(x_{m}(i)|H_{0})\\ & =\frac{1}{{\left(2\pi \sigma_{n}^{2}\right)}^{\frac{M(N_{c}+N_{d})}{2}}}\exp(-\frac{{\left\|x\right\|}^{2}}{2\sigma_{n}^{2}}) \end{array}$$

On the other hand, the p.d.f *p*(**x**) under *H*_1_ can be expressed as

(8) $$p(x|{\text{H}}_{1})=\frac{1}{{\left(2\pi \right)}^{\frac{M(N_{c}+N_{d})}{2}}{\left|B_{\tau }\right|}^{\frac{1}{2}}}\exp(-\frac{1}{2}x^{H}B_{\tau }^{-1}x)$$

where **B***_τ_* is the covariance matrix of x, and its structure dependents on timing delay *τ*. Then using Equations (7) and (8), the DC-OFDM detector can be expressed as:

(9) $$T_{Dc-\text{OFDM}}=\frac{{p^{\left(n\right)}(x|\theta )|}_{\theta =0}}{p(x|0)}\overset{H_{0}}{\underset{H_{1}}{≶}}\gamma_{DC-\text{OFDM}}$$

In order to get more specific expression of Equation (8) and prepare for the derivation processes, we rewrite it according to the structure of **B***_τ_*. Moreover, since the value of *τ* decides the structure of **B***_τ_*, we tend to discuss *τ* to get the different test statistics.

### 3.1. Timing Delay Is Smaller Than the Length of CP

When *τ* ∈ [0, *N_c_*], we get the expression of ${\text{x}}^{H}B_{\tau }^{-1}\text{x}$ that

(10) $$\begin{array}{l} \begin{array}{l} x^{H}B_{\tau }^{-1}x\\ =\overset{M-1}{\underset{m=0}{\text{∑}}}\overset{N_{c}-\tau -1}{\underset{i=0}{\text{∑}}}x_{m}(i)*(\frac{\sigma_{n}^{2}+\theta^{2}}{2\theta^{2}\sigma_{n}^{2}+\sigma_{n}^{4}}{\text{x}}_{m}(i)-\frac{\theta^{2}}{2\theta^{2}\sigma_{n}^{2}+\sigma_{n}^{4}}x_{m}(i+N_{d}))\\ +\overset{N_{c}+N_{d}-\tau -1}{\underset{i=N_{d}}{\text{∑}}}x_{0}(i)*(\frac{\sigma_{n}^{2}+\theta^{2}}{2\theta^{2}\sigma_{n}^{2}+\sigma_{n}^{4}}x_{0}(i)-\frac{\theta^{2}}{2\theta^{2}\sigma_{n}^{2}+\sigma_{n}^{4}}x_{0}(i-N_{d}))\\ +\overset{M-2}{\underset{m=0}{\text{∑}}}\overset{N_{c}+N_{d}-1}{\underset{i=N_{c}+N_{d}-\tau }{\text{∑}}}x_{m}(i)*(\frac{\sigma_{n}^{2}+\theta^{2}}{2\theta^{2}\sigma_{n}^{2}+\sigma_{n}^{4}}x_{m}(i)-\frac{\theta^{2}}{2\theta^{2}\sigma_{n}^{2}+\sigma_{n}^{4}}x_{m}(i+N_{d}))\\ +\overset{M-1}{\underset{m=1}{\text{∑}}}[\overset{N_{c}+N_{d}-\tau -1}{\underset{i=N_{d}-\tau }{\text{∑}}}x_{m}(i)*(\frac{\sigma_{n}^{2}+\theta^{2}}{2\theta^{2}\sigma_{n}^{2}+\sigma_{n}^{4}}x_{m}(i)-\frac{\theta^{2}}{2\theta^{2}\sigma_{n}^{2}+\sigma_{n}^{4}}x_{m}(i-N_{d}))\\ +\overset{N_{d}-\tau -1}{\underset{N_{c}-\tau }{\text{∑}}}\frac{1}{\theta^{2}+\sigma_{n}^{2}}{\left|x_{m}(i)\right|}^{2}]+\overset{N_{d}-1}{\underset{i=N_{c}-\tau }{\text{∑}}}\frac{1}{\theta^{2}+\sigma_{n}^{2}}{\left|x_{0}(i)\right|}^{2}+\overset{N_{c}+N_{d}-1}{\underset{i=N_{c}+N_{d}-\tau }{\text{∑}}}\frac{1}{\theta^{2}+\sigma_{n}^{2}}{\left|x_{M-1}(i)\right|}^{2} \end{array} \end{array}$$

Furthermore, |**B***_τ_*| will also be obtained that

(11) |Bτ|=σn2M(Nc+Nd)(1+θ2σn2)(M−1)(Nd−Nc)+2τ×(1+2θ2σn2)(M−1)(Nd−Nc)−τ

so Equation (8) can be written as

(12) $$p(x|\theta )=\frac{1}{{\left(2\pi \sigma_{n}^{2}\right)}^{\frac{M(N_{c}+N_{d})}{2}}}A_{1}\exp(-\frac{1}{2}A_{2})$$

where

$$\begin{array}{ll} A_{1} & ={\left(1+\frac{\theta^{2}}{\sigma_{n}^{2}}\right)}^{-\frac{(M-1)(N_{d}-N_{c})+2\tau }{2}}{\left(1+2\frac{\theta^{2}}{\sigma_{n}^{2}}\right)}^{-\frac{(M-1)(N_{d}-N_{c})-\tau }{2}}\\ A_{2} & ={\text{x}}^{H}B_{\tau }^{-1}\text{x} \end{array}$$

Therefore, the first derivative of *p*(**x**|*θ*) is

(13) $$\begin{array}{ll} p^{\left(1\right)}(x|\theta ) & =\frac{1}{{\left(2\pi \sigma_{n}^{2}\right)}^{\frac{M(N_{c}+N_{d})}{2}}}[A_{1}^{\left(1\right)}\exp(-\frac{1}{2}A_{2})\\ & -\frac{1}{2}A_{1}\exp(-\frac{1}{2}A_{2})A_{2}^{\left(1\right)}] \end{array}$$

where $A_{1}^{\left(1\right)}$ and $A_{2}^{\left(1\right)}$ are the first derivative of *A*_1_ and *A*_2_. Since the expressions of $A_{1}^{\left(1\right)}$ and $A_{2}^{\left(1\right)}$ are too complex, the details are shown in Equations (A1) and (B1) in Appendix, respectively.

As is illustrated in Equations (A1) and (B1), $A_{1}^{\left(1\right)}$ and $A_{2}^{\left(1\right)}$ at *θ* = 0 are equal to zero and so is *p*^(1)^ (**x**|*θ*) at *θ* = 0. Therefore, higher derivative have to be computed. The second derivative of *p*(**x**|*θ*) is

(14) $$\begin{array}{ll} p^{\left(2\right)}(x|\theta ) & =\frac{1}{{\left(2\pi \sigma_{n}^{2}\right)}^{\frac{M(N_{c}+N_{d})}{1}}}A_{1}^{\left(2\right)}\exp(-\frac{1}{2}A_{2})\\ & -\frac{1}{2}A_{1}^{\left(1\right)}\exp(-\frac{1}{2}A_{2})A_{2}^{\left(1\right)}-\frac{1}{2}A_{1}^{\left(1\right)}\exp(-\frac{1}{2}A_{2})A_{2}^{\left(1\right)}\\ & +\frac{1}{4}A_{1}\exp(-\frac{1}{2}A_{2}){\left(A_{2}^{\left(1\right)}\right)}^{2}-\frac{1}{2}A_{1}\exp(-\frac{1}{2}A_{2})A_{2}^{\left(2\right)}] \end{array}$$

For the $A_{1}^{\left(2\right)}$ and $A_{2}^{\left(2\right)}$, we get them from $A_{1}^{\left(1\right)}$ and $A_{2}^{\left(1\right)}$. When *θ* = 0, $A_{1}^{\left(2\right)}$ and $A_{1}^{\left(2\right)}$ will be equal to

(15) $$A_{1}^{\left(2\right)}=-\frac{1}{\sigma_{n}^{2}}(M-1)(N_{c}+N_{d})$$

and

(16) $$\begin{array}{ll} A_{2}^{\left(2\right)} & =-\frac{2}{\sigma_{n}^{4}}[\overset{M-1}{\underset{m=0}{\text{∑}}}\overset{N_{c}+N-d-1}{\underset{i=0}{\text{∑}}}{\left|x_{m}(i)\right|}^{2}+\overset{M-1}{\underset{m=0}{\text{∑}}}\overset{N_{c}-\tau -1}{\underset{i=0}{\text{∑}}}x_{m}(i)*x_{m}(i+N_{d})\\ & +\overset{M-2}{\underset{m=0}{\text{∑}}}\overset{N_{c}+N_{d}-1}{\underset{N_{c}+N_{d}-\tau }{\text{∑}}}x_{m}(i)*x_{m}(i+N_{d})+\overset{M-1}{\underset{m=1}{\text{∑}}}\overset{N_{c}+N-\tau -1}{\underset{i=N_{d}-\tau }{\text{∑}}}x_{m}(i)*x_{m}(i-N_{d})\\ & +\overset{N_{c}+N_{d}-\tau -1}{\underset{i=N_{d}}{\text{∑}}}x_{M-1}(i)*x_{M-1}(i-N_{d})] \end{array}$$

So, according to Equations (12), (14)–(16), we get the test statistic under *τ* ∈ [0, *N_c_*] that

(17) TDC−OFDM1=p(2)(x|θ=0)p(x|0)=A1(2)−12A2(2)=1σn4[∑m=0M−1∑i=0Nc+Nd−1|xm(i)|2+∑m=0M−1∑i=0Nc−τ−1xm(i)*xm(i+Nd)+∑m=0M−2∑i=Nc+Nd−1Nc+Nd−1xm(i)*xm(i+Nd)+∑m=1M−1∑i=Nd−τNc+Nd−τ−1xm(i)*xm(i−Nd)+∑i=NdNc+Nd−τ−1xM−1(i)*xM−1(i−Nd)−σn2(M−1)(Nc+Nd)]≶H1H0γDC−OFDM1

where *γ_DC_*_−_*_OFDM_*_1_ is the threshold when *τ* ∈ [0, *N*_c_].

### 3.2. Timing Delay Is Equal to Other Values

When *τ* ∈ [*N_c_* + 1, *N_d_* − 1] and *τ* ∈ [*N_d_*, *N_c_*+ *N_d_* − 1], since the processes of derivation of the test statistics are the same as described above, we will not give the details here due to lack of space. Then, the test statistics *T_DC_*_−_*_OFDM_*_2_ and *T_DC_*_−_*_OFDM_*_3_ under *τ* ∈ [*N_c_* +1, *N_d_* − 1] and *τ* ∈ [*N_d_*, *N_c_* + *N_d_* − 1] are

(18) TDC−OFDM2=p(2)(x|θ=0)p(x|0)=1σn4[∑m=0M−1∑i=0Nc+Nd−1|xm(i)|2+∑m=0M−2∑i=Nc+Nd−τNc+Nd−τ−1xm(i)*xm(i+Nd)+∑m=1M−1∑i=Nd−τNc+Nd−τ−1xm(i)*xm(i−Nd)−σn2(M−1)(Nc+Nd)]≶H1H0γDC−OFDM2

and

(19) TDC−OFDM3=p(2)(x|θ=0)p(x|0)=1σn4[∑m=0M−1∑i=0Nc+Nd−1|xm(i)|2+∑m=0M−2∑i=Nc+nd−τ2Nc+nd−τ−1xm(i)*xm(i+Nd)+∑m=0M−1∑i=Nc+2Nd−τNc+Nd−1xm(i)*xm(i−Nd)+∑m=1M−1∑i=0Nc+Nd−τ−1xm(i)*xm(i−Nd)+∑i=Nc+Nd−τNc−1xM−1(i)*xM−1(i−Nd)−σn2(M−1)(Nc+Nd)]≶H1H0γDC−OFDM3

where the *γ_DC_*_−_*_OFDM_*_2_ and *γ_DC_*_−_*_OFDM_*_3_ are the thresholds when *τ* ∈ [*N_c_* + 1, *N_d_* − 1] and *τ* ∈ [*N_d_*, *N_c_* + *N_d_* − 1], respectively. As mentioned above, we get test statistics of DC-OFDM detector under different *τ* according to Equations (17)–(19). However, we can see from these equations that it is not easy to get the closed-form expressions for the distribution of the test statistics, so we will discuss it in the next section.

The DC-OFDM detector is a suboptimal detector to sense the OFDM signal by taking advantage of differential operations to get the locally optimal solution. Moreover, it provides us a new way to improve the traditional detectors based on employing differential operations. In the next section, we will give the examples of applying the merits of differential characteristics to improve the traditional CP detector and PT detector.

## 4. DC-CP Detection and DC-PT Detection

In this section, we will discuss new CP detector and PT detector based on the advantages of structure of OFDM signal by using the auto-correlation of CP and PT. Through the differential operation, the performance of traditional CP detector and PT detector will be improved. Here we also take interest of testing *θ* = 0 against *θ* > 0. Moreover, the computation complexity of the proposed algorithms will be analyzed and compared with other approaches.

### 4.1. DC-CP Sensing Algorithm

Considering the unique feature of the CP, we propose the following measure of auto-correlation, and apply the signal model in Equation (2):

(20) $$\begin{array}{ll} R_{i} & =\sum_{m=0}^{M-1}x_{m}^{*}(i)x_{m}(i+N_{d})\\ & =\sum_{m=0}^{M-1}\left(hs_{m}(i-\tau )+e_{m}(i))*(hs_{m}(i-\tau +N_{d})+e_{m}(i+N_{d})\right) \end{array}$$

where * stands for the complex conjugate operation, i*i* = 0,…, *N_c_* + *N_d_* − 1, and *θ* = |*h*|. According to the central limit theorem, if the *M* is large enough, *R_i_* could approximate to a complex Gaussian random variable. So according to Equation (20), the mean of *R_i_* can be computed as

(21) $$\mu_{\text{i}}=\text{E}[{\text{R}}_{i}|\theta ]=M\theta^{2}\text{E}[s_{m}^{*}(i-\tau )s_{m}(i-\tau +N_{d})]$$

Let *C* = [1, 2,…, *N_c_*], so when *i* − *τ* ∈ *C*, we get

(22) $$\text{E}[s_{m}^{*}(i-\tau )s_{m}(i-\tau +N_{d})]=E_{s}=1$$

Let us define the indicator function

(23) $$1_{C}(k):=\{\begin{array}{ll} 1, & \text{if}\;k\in C\\ 0, & \text{if}\;k\notin C \end{array}$$

then applying Equations (22) and (23), we can rewrite Equation (21) as

(24) $$\mu_{i}=\theta^{2}M1_{C}(i-\tau )$$

Now, the variance of *R_i_* can be computed as

(25) $$\sigma_{\text{i}}^{2}=\theta^{4}1_{C}^{2}(i-\tau )M+M{\left(\theta^{2}+\sigma_{n}^{2}\right)}^{2}$$

In this paper we intend to employ ${\left\{R_{i}\right\}}_{i=0}^{N_{c}+N_{d}-1}$, rather than a single *R_i_*, so we define the vector:

(26) $$R=[R_{0},R_{1},\ldots ,R_{N_{c}+N_{d}-1}]$$

To simplify the derivation of the joint distribution, we assume that all the *R_i_* in Equation (26) are independent. We can define the following test statistic based on the Equation (6):

(27) $$T_{DC-CP}=\frac{{p^{\left(n\right)}(R|\theta )|}_{\theta =0}}{p^{\left(n\right)}(R|0)}$$

where *p*(**R**|*θ*) is p.d.f of **R** and

(28) $$p(\text{R}|\theta )=\prod_{i=0}^{N_{c}+N_{d}-1}p(R_{i}|\theta )$$

*R_i_* is Gaussian distribution, so

(29) $$p(R_{i}|\theta )=\frac{1}{\sqrt{2\pi }}D_{1}\exp(D_{2})$$

where

(30) $$D_{1}={\left(\theta^{4}1_{C}^{2}(i-\tau )M+M\left({\left(\theta^{2}+\sigma_{n}^{1}\right)}^{2}\right)\right)}^{-\frac{1}{2}}$$

and

(31) $$D_{2}=\frac{{\left|R_{i}-\theta^{2}M1_{C}(i-\tau )\right|}^{2}}{2(\theta^{4}1_{C}^{2}(i-\tau )M+M{\left(\theta^{2}+\sigma_{n}^{2}\right)}^{2})}$$

The first derivative of *p*(**R**|*θ*) is

(32) $$p^{\left(1\right)}(\text{R}|\theta )=\sum_{i=0}^{N_{c}+N_{d}-1}p^{\left(1\right)}(R_{i}|\theta )\prod_{\begin{array}{c} \text{j}=0\\ j\neq i \end{array}}^{N_{c}+N_{d}-1}p(R_{j}|\theta )$$

and

(33) $$p^{\left(1\right)}(R_{i}|\theta )=\frac{1}{\sqrt{2\pi }}(D_{1}^{\left(1\right)}\exp(D_{2})+D_{1}\exp(D_{2})(D_{2}^{1}))$$

where $D_{1}^{\left(1\right)}$ and $D_{2}^{\left(1\right)}$ are the first derivative of *D*_1_ and *D*_2_, and the specific expressions are shown in section Appendix. When *θ* = 0, $D_{1}^{\left(1\right)}$ and $D_{2}^{\left(1\right)}$ are both equal to zero. So *p*^(1)^(**R**|*θ*) = 0 at *θ* = 0. Therefore, we have to compute higher derivatives.

The second derivative of *p*(**R**|*θ*) is

(34) $$\begin{array}{ll} p^{\left(2\right)}(\text{R}|\theta ) & =\sum_{i=0}^{N_{c}+N_{d}-1}\left[p^{\left(2\right)}(R_{i}|\theta \right)\prod_{\begin{array}{c} j=0\\ j\neq i \end{array}}^{N_{c}+N_{d}-1}p(R_{j}|\theta )]\\ & +\sum_{i=0}^{N_{c}+N_{d}-1}\left[p^{\left(1\right)}(R_{i}|\theta \right)\sum_{\begin{array}{c} j=0\\ j\neq i \end{array}}^{N_{c}+N_{d}-1}\left(p^{\left(1\right)}(R_{j}|\theta \right)\prod_{\begin{array}{c} k=0\\ k\neq 1\\ k\neq j \end{array}}^{N_{c}+N_{d}-1}p(R_{k}|\theta ))] \end{array}$$

and

(35) $$p^{\left(2\right)}({\text{R}}_{i}|\theta )=\frac{1}{\sqrt{2\pi }}\left(D_{1}^{\left(2\right)}\exp(D_{2})+D_{1}^{\left(1\right)}\exp(D_{2})D_{2}^{\left(1\right)}+D_{1}^{\left(1\right)}\exp(D_{2})D_{2}^{\left(1\right)}+D_{1}\exp(D_{2}){\left(D_{2}^{\left(1\right)}\right)}^{2}+D_{1}\exp(D_{2})D_{2}^{\left(2\right)}\right)$$

The expressions of $D_{1}^{\left(2\right)}$ and $D_{2}^{\left(2\right)}$ are also given in Appendix. So when *θ* = 0, we get that

(36) $$D_{1}^{\left(2\right)}(\theta =0)=\frac{2M\sigma_{n}^{2}}{{\left(M\sigma_{n}^{4}\right)}^{\frac{3}{2}}}$$

and

(37) D2(2)(θ=0)=2Re(Ri)M1C(i−τ)Mσn4+2Mσn2|Ri|2(Mσn4)2

then Equation (27) could be written as

(38) TDC−CP=p(2)(R|θ)|θ=0p(R|0)=∑i=0Nc+Nd−1[2Re(Ri)1C(i−τ)σn4+2|Ri|2Mσn6−2σn2]≶H1H0γDC−CP

### 4.2. DC-PT Sensing Algorithm

DC-PT detector is another typical example of employing differential operation to improve the traditional detectors. Since the specific derivation process of the DC-PT algorithm is similar to DC-CP algorithm, we will briefly introduce the DC-PT sensing algorithm.

For an OFDM system, in order to achieve the satisfied estimating results, PTs are usually employed. In this paper, we only consider the circular configuration that the pilot subcarrier indexes cyclically changes to avoid lengthiness. To utilize PT to sense the signal, we first need to compute FFT at the receiver to convert the signals from time-domain to frequency domain, and then compute the correlation of pilot symbols. The received signal after removing CP and FFT process can be expressed as

(39) $$\begin{array}{ll} {\tilde{y}}_{m}(\text{n}) & =\frac{1}{\sqrt{N}}\sum_{l=0}^{N_{d}-1}\sqrt{\frac{1}{N_{d}}}\sum_{k=0}^{N_{d}-1}H_{k}I_{m}(k)e^{j2\pi lk/N_{d}}e^{-j2\pi \ln/N_{d}}+\frac{1}{\sqrt{N}}\sum_{l=0}^{N_{d}-1}e_{m}(\text{n})e^{-j2\pi \ln/N_{d}}\\ & =H_{\text{n}}I_{m}(n)+{\text{W}}_{m}(n) \end{array}$$

where *H_n_* = *he*^−^*^j^*^2^*^πn/Nd^* is the channel gain of subcarrier *n*. *W_m_*(*n*) is frequency-domain noise with zero mean and $\sigma_{n}^{2}$ variance. We assume that there are *N_p_* PT in each block with the same amplitude, and the pilots are the same every several blocks but different among one block. So two OFDM blocks with block index difference *kt* have the same PT arrangement, where *k* = 1,2,…, and *t* = *m* − *u*(1 ≤ *m* ≤ *M*, 1 ≤ *u* ≤ *M*) stands for the block index difference. Employing the feature of PT, this detector of OFDM signal can be written as [21]:

(40) $$G_{t}=\sum_{N_{t}}\sum_{n=0}^{N_{p-1}}{\tilde{y}}_{m}(n){\tilde{y}}_{u}(n)*$$

where *N_t_* is the number of block pairs (*m*, *u*) with the same PT order. The p.d.f of *G_t_* under *H*_0_ and *H*_1_ can be computed as:

(41) $$\begin{array}{l} G_{t}|H_{0}\sim CN(0,N_{t}N_{p}\sigma_{n}^{4}),\\ G_{t}|H_{1}\sim CN(N_{t}N_{p}\theta^{2},N_{t}N_{p}\sigma_{n}^{4}+2N_{t}N_{p}\sigma_{n}^{2}) \end{array}$$

where *θ* = |*h*|. Thus we pupose the DC-PT test statistic based on the Equation (6):

(42) $$T_{DC-PT}=\frac{{p^{\left(n\right)}(G_{t}|0)|}_{\theta =0}}{p(G_{t}|0)}$$

where *p*(*G_t_*|) is the p.d.f of *G_t_*. Based on Equation (41), using mathematica and some algebra, the test statistic of DC-PT detector can be expressed as

(43) TDC−PT=p(2)(Gt|θ)|θ=0p(Gt|0)=2Re(Gt)σn4+2|Gt|2NlNpσn6−2σn2≶H1H0γDC−PT

## 5. Probabilities of Misdetection and False Alarm

In the previous sections, we have derived the test statistics of the three proposed methods. In this section, we will apply some reasonable assumptions and approximations to get specific expressions for the probabilities of misdetection and false alarm of the proposed sensing approaches.

### 5.1. P_m_ and P_f_ of DC-OFDM Detector

As is discussed in Section “Algorithm of DC-OFDM Detection”, the test statistic of DC-OFDM detector depends on the time delay *τ*. So, it would be valuable to discuss the detector under different *τ*. For the reason that the structures of test statistics for different *τ* are similar, we just give the details when *τ* ∈ [*N_c_* + 1, *N_d_* − 1]. For other situations, we will directly provide the results.

When *τ* ∈ [*N_c_* + 1, N*_d_* − 1], the test statistic is shown in Equation (18), which mainly contains four parts. So Equation (18) can be rewritten as

(44) $$\begin{array}{ll} T_{DC-\text{OFDM}2} & =\frac{p^{\left(2\right)}(\text{x}|\theta =0)}{p(\text{x}|0)}\\ & =\frac{1}{\sigma_{n}^{4}}[I_{1}+I_{2}+I_{3}-\sigma_{n}^{2}(M-1)(N_{c}+N_{d})] \end{array}$$

where

$$\begin{array}{ll} I_{1} & =\overset{M-1}{\underset{m=0}{\text{∑}}}\overset{N_{c}+N_{d}-1}{\underset{i=0}{\text{∑}}}{\left|x_{m}(i)\right|}^{2}\\ I_{2} & =\overset{M-2}{\underset{m=0}{\text{∑}}}\overset{2N_{c}+N_{d}-\tau -1}{\underset{i=N_{c}+N_{d}-\tau }{\text{∑}}}x_{m}(i)*x_{m}(i+N_{d})\\ I_{3} & =\overset{M-1}{\underset{m=1}{\text{∑}}}\overset{N_{c}+N_{d}-\tau -1}{\underset{i=N_{d}-\tau }{\text{∑}}}x_{m}(i)*x_{m}(i-N_{d}) \end{array}$$

For an OFDM system, a small communication time will result in a great number of transmitted samples. So according to the central limit theory, *T_DC_*_−_*_OFDM_*_2_ is approximate to complex Gaussian distribution, under both *H*_0_ and *H*_1_:

(45) $$\begin{array}{l} T_{DC-\text{OFDM}2}|H_{0}\sim CN(\mu_{T,0},\sigma_{T,0}^{2})\\ T_{DC-\text{OFDM}2}|H_{1}\sim CN(\mu_{T,1},\sigma_{T,1}^{2}) \end{array}$$

Now we need to compute both the means and variances of *T_DC_*_−_*_OFDM_*. The mean of *T_DC_*_−_*_OFDM_* is

(46) $$\begin{array}{ll} \mu_{T} & =\text{E}[\frac{1}{\sigma_{n}^{4}}(I_{1}+I_{2}+I_{3}-\sigma_{n}^{2}(M-1)(N_{c}+N_{d}))]\\ & =\frac{1}{\sigma_{n}^{4}}[\mu_{I_{1}}+\mu_{I_{2}}+\mu_{I_{3}}-\sigma_{n}^{2}(M-1)(N_{c}+N_{d}) \end{array}$$

where *μ_I_i__* is the mean of *I_i_*, *i* = 1, 2, 3. The details of *μ_I_i__* are given in the Appendix. The results are listed as

(47) $$\begin{array}{ll} \mu_{T,0} & =\frac{1}{\sigma_{n}^{2}}(N_{c}+N_{d})\\ \mu_{T,1} & =\frac{1}{\sigma_{n}^{4}}[((3M-2)N_{c}+MN_{d})\sigma_{s}^{2}+(N_{c}+N_{d})\sigma_{n}^{2}] \end{array}$$

The variance of *T_DC_*_−_*_OFDM_*_2_ can be computed as

(48) $$\begin{array}{ll} \sigma_{T}^{2} & =\text{D}[\frac{1}{\sigma_{n}^{4}}({\text{I}}_{1}+{\text{I}}_{2}+{\text{I}}_{3}-\sigma_{n}^{2}(\text{M}-1)({\text{N}}_{c}+{\text{N}}_{d}))]\\ & =\frac{1}{\sigma_{n}^{8}}[(\sigma_{I_{1}}^{2}+\sigma_{I_{2}}^{2}+\sigma_{I_{2}}^{2}+2(\text{COV}(I_{1}\;I_{2})+\text{COV}(I_{1}\;I_{3})+\text{COV}(I_{2}\;I_{3}))] \end{array}$$

where $\sigma_{I_{i}}^{2}$ is the variance of *I_i_*, *i* = 1, 2, 3; COV (*I_i_I_j_*) stands for the covariance of *I_i_* and *I_j_*. Again, the derivation processes are shown in the Appendix. Thus, the variances of *T_DC_*_−_*_OFDM_*_2_ under *H*_0_ and *H*_1_ are:

(49) $$\begin{array}{ll} \sigma_{T,0}^{2} & =\frac{1}{\sigma_{n}^{4}}((3M-2)N_{c}+MN_{d})\\ \sigma_{T,1}^{2} & =\frac{1}{\sigma_{n}^{8}}[((3M-1)N_{c}+MN_{d}){\left(\sigma_{s}^{2}+\sigma_{n}^{2}\right)}^{2}+8MN_{c}(\sigma_{s}^{2}+\sigma_{n}^{2})\sigma_{s}^{2}] \end{array}$$

Therefore, the probabilities of false alarm and misdetection can be easily expressed as

(50) $$\begin{array}{l} P_{f}=Q\left(\frac{\gamma T_{DC-\text{OFDM}2}-\mu_{T,0}}{\sqrt{\sigma_{T,0}^{2}}}\right)=0\left(\frac{\gamma T_{DC-\text{OFDM}2}-\frac{1}{\sigma_{n}^{2}}(N_{c}+N_{d})}{\sqrt{\frac{1}{\sigma_{n}^{2}}((3M-2)N_{c}+MN_{d})}}\right)\\ P_{m}=1-Q\left(\frac{\gamma T_{DC-\text{OFDM}2}-\mu_{T,1}}{\sqrt{\sigma_{T,1}^{2}}}\right)\\ =1-Q\left(\frac{\gamma T_{DC-\text{OFDM}2}-\frac{1}{\sigma_{n}^{4}}[((3M-2)N_{c}+MN_{d})\sigma_{s}^{2}+(N_{c}+N_{d})\sigma_{n}^{2}]}{\sqrt{\frac{1}{\sigma_{n}^{8}}[((3M-1)N_{c}+MN_{d}){\left(\sigma_{s}^{2}-\sigma_{n}^{2}\right)}^{2}+8MN_{c}(\sigma_{s}^{2}+\sigma_{n}^{2})\sigma_{s}^{2}]}}\right) \end{array}$$

Generally speaking, we will calculate the *P_m_* for a fixed *P_f_* to test the performance the spectrum sensing method. Thus the threshold according to the Equation (50) is

(51) $$\gamma T_{DC-\text{OFDM}2}=\sqrt{\sigma_{T,1}^{2}}Q^{-1}(P_{f})+\frac{1}{\sigma_{n}^{2}}(N_{c}+N_{d})$$

Employing the same method, it is easy to get the *P_f_* and *P_m_* when *τ* ∈ [0, *N_c_*] and *τ* ∈ [*N_d_, N_c_* + *N_d_* − 1]. The results are listed as

(52) $$\{\begin{array}{l} P_{f}=Q\left(\frac{\gamma T_{DC-\text{OFDM}1}-\frac{1}{\sigma_{n}^{2}}(N_{c}+N_{d})}{\sqrt{\frac{1}{\sigma_{n}^{4}}((3M-2)N_{c}+MN_{d}}}\right)\\ P_{m}=1-Q\left(\frac{\gamma T_{DC-\text{OFDM}1}-\frac{1}{\sigma_{n}^{4}}[((3M-2)N_{c}+MN_{d})\sigma_{s}^{4}+(N_{c}+N_{d})\sigma_{n}^{2}]}{\sqrt{\frac{1}{\sigma_{n}^{8}}[((3M-1)N_{c}+MN_{d}){\left(\sigma_{s}^{2}+\sigma_{n}^{2}\right)}^{2}+8MN_{c}(\sigma_{s}^{2}+\sigma_{n}^{2})\sigma_{s}^{2}]}}\right),\tau \in [0,N_{c}]\\ \gamma T_{DC-\text{OFDM}1}=\sqrt{\sigma_{T,1}^{2}}Q^{-1}(P_{f})+\frac{1}{\sigma_{n}^{2}}(N_{c}+N_{d}) \end{array}$$

(53) $$\{\begin{array}{l} P_{f}=Q\left(\frac{\gamma T_{DC-\text{OFDM}3}-\frac{1}{\sigma_{n}^{2}}(N_{c}+N_{d})}{\sqrt{\frac{1}{\sigma_{n}^{4}}((3M-2)N_{c}+MN_{d}}}\right)\\ P_{m}=1-Q\left(\frac{\gamma T_{DC-\text{OFDM}3}-\frac{1}{\sigma_{n}^{4}}[((3M-2)N_{c}+MN_{d})\sigma_{s}^{4}+(N_{c}+N_{d})\sigma_{n}^{2}]}{\sqrt{\frac{1}{\sigma_{n}^{8}}[((3M-1)N_{c}+MN_{d}){\left(\sigma_{s}^{2}+\sigma_{n}^{2}\right)}^{2}+8MN_{c}(\sigma_{s}^{2}+\sigma_{n}^{2})\sigma_{s}^{2}]}}\right),\tau \in [N_{d},N_{c}+N_{d}-1]\\ \gamma T_{DC-\text{OFDM}3}=\sqrt{\sigma_{T,1}^{2}}Q^{-1}(P_{f})+\frac{1}{\sigma_{n}^{2}}(N_{c}+N_{d}) \end{array}$$

### 5.2. P_m_ and P_f_ of DC-PT Detector

In order to get the cumulative distribution function of DC-PT detector, we need to rewrite Equation (43) in an equivalent format as

(54) T′DC−PT=(Re(Gt)+NtNpσn22+(Im(Gt))2≶H1H0γ′DC−PT

where Im(.) takes the imaginary part, and the corresponding threshold ${\gamma^{\prime }}_{DC-PT}=\frac{N_{t}N_{p}\sigma_{n}^{6}\gamma_{DC-PT}}{2}+N_{t}N_{p}\sigma_{n}^{4}+\frac{N_{t}^{2}N_{p}^{2}\sigma_{n}^{4}\gamma_{DC-PT}}{4}$. For *G_t_* is complex Gaussian distribution, it is easy to know a=Re(Gt)+NtNpσn22∼N(μa,σa2), b=Im(Gt)∼N(μb,σb2). *μ_a_*, *μ_b_*, $\sigma_{a}^{2}$ and $\sigma_{b}^{2}$ are the means and variances of *a* and *b*, respectively. And $\sigma_{a}^{2}=\sigma_{b}^{2}=\frac{1}{2}\sigma_{G_{t}}^{2}$. Since the *a* and *b* are Gaussian-distributed random variables, so ${\left(a\right)}^{2}/\left(\sigma_{G_{t}}^{2}/2\right)$ and ${\left(b\right)}^{2}/\left(\sigma_{G_{t}}^{2}/2\right)$ are followed noncentral chi-square distribution with one degree of freedom. Moreover, the non-centrality parameters are $\lambda_{a}=2\mu_{a}^{2}/\sigma_{G_{t}}^{2}$, $\lambda_{b}=2\mu_{b}^{2}/\sigma_{G_{t}}^{2}$, respectively.

As is well known, the sum of independent chi-square distributed random variables *z*_1_, *z*_2_, …, *z_n_* still obeys noncentral chi-square distribution. The non-centrality parameter and the degrees of freedom are $\sum_{i=1}^{N}\lambda_{i}$ and $\sum_{i=1}^{N}k_{i}$, where λ*_i_* and *k_i_* are the non-centrality parameter and the degree of freedom of z*_i_*. As the result, ${T^{\prime }}_{DC-PT}/\left(\sigma_{G_{t}}^{2}/2\right)=\left(a^{2}+b^{2}\right)/\left(\sigma_{G_{t}}^{2}/2\right)$ is noncentral chi-square distributed with non-centrality parameter λ = λ*_a_* + λ*_b_* and two degrees of freedom.

According to Equation (41), under *H*_0_, the *G_t_* has variance $N_{t}N_{p}\sigma_{n}^{4}$ with zero mean, which indicates that $\mu_{a}=\frac{N_{t}N_{p}\sigma_{n}^{2}}{2}$, *μ_b_* = 0. On the other hand, under *H*_1_, $\mu_{a}=\frac{N_{t}N_{p}\theta^{2}+N_{t}N_{p}\sigma_{n}^{2}}{2}$, *μ_b_* = 0. Applying these results, the non-centrality parameter λ under *H*_0_ and *H*_1_ can be expressed as

(55) $$\begin{array}{ll} \lambda_{DC-PT,0} & =\frac{N_{t}N_{p}}{2},\\ \lambda_{DC-PT,1} & =\frac{N_{t}N_{p}{\left(2\theta^{2}+\sigma_{n}^{2}\right)}^{2}}{2\left(\sigma_{n}^{4}+2\theta^{2}+\sigma_{n}^{2}\right)} \end{array}$$

Based on the conclusions above, the *P_f_, P_m_* and the threshold of DC-PT detector can be written as

(56) $$\begin{array}{l} P_{f}=1-F\left({\gamma^{\prime }}_{DC-PT}/(\sigma_{G_{t},0}^{2}/2);2,\lambda_{DC-PT,0}\right)\\ P_{m}=F\left({\gamma^{\prime }}_{DC-PT}/(\sigma_{G_{t},1}^{2}/2);2,\lambda_{DC-PT,1}\right)\\ {\gamma^{\prime }}_{DC-PT}=\frac{\sigma_{G_{t},0}^{2}}{2}F^{-1}(1-P_{f};2,\lambda_{DC-PT,0}) \end{array}$$

where *F*(.) is the cumulative distribution function of non-central chi-square distributed random variable; $\sigma_{G_{t},0}^{2}$ and $\sigma_{G_{t},1}^{2}$ are the variances of *G_t_* under *H*_0_ and *H*_1_, respectively.

### 5.3. P_m_ and Pf of DC-CP Detector

On the basis of the definitions and conclusions in the previous section, the *P_m_* and *P_f_* of DC-CP detector can be achieved by employing the similar approach. According to Equation (38) the test statistic of DC-CP detector can be rewritten as

(57) T′DC−CP=∑i=0Nc+Nd−1(Re(Ri)+1C(i−τ)Mσn22)2+(Im(Ri))2=∑i=0Nc+Nd−1αi2+βi2≶H1H0γ′DC−CP

where ${\gamma^{\prime }}_{DC-CP}=\frac{M\sigma_{n}^{6}\gamma_{DC-CP}}{2}+\sum_{i=0}^{N_{c}+N_{d}-1}\left(\frac{1c{\left(i-r\right)}^{2}M+4}{2\sigma_{n}^{2}}\right)$; αi=Re(Ri)+1c(i−r)Mσn22 and *β_i_* = IM(*R_i_*), so ${T^{\prime }}_{DC-CP}$ contains *N_c_* + *N_d_* pairs of *α*^2^ and *β*^2^. Since *R_i_* is complex Gaussian random variable, $\alpha_{i}∼N\left(\mu_{\alpha_{i}},\sigma_{i}^{2}/2\right)$, $\beta_{i}∼N\left(\mu_{\beta_{i}},\sigma_{i}^{2}/2\right)$, where $\sigma_{i}^{2}$ is the variance of *R_i_*. It is easy to proof that the *α* and *β* are independent, and the distribution of $\alpha_{i}^{2}/\left(\sigma_{i}^{2}/2\right)$ and $\beta_{i}^{2}/\left(\sigma_{i}^{2}/2\right)$ are noncentral chi-square distributed with one degree of freedom and non-centrality parameters are $\lambda_{\alpha i}=2\mu_{\alpha_{i}}^{2}/\sigma_{i}^{2}$, $\lambda_{\beta i}=2\mu_{\beta_{i}}^{2}/\sigma_{i}^{2}$.

**Figure 3:**
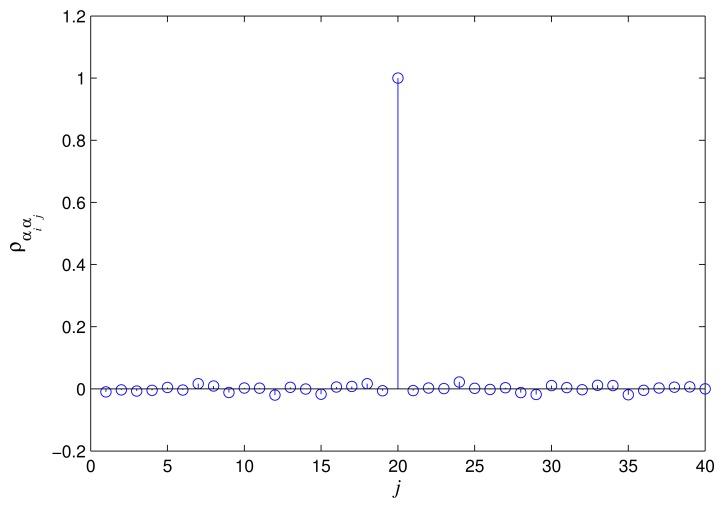
The correlation coefficient *ρ_αiαj_* when *i* = 20.

**Figure 4:**
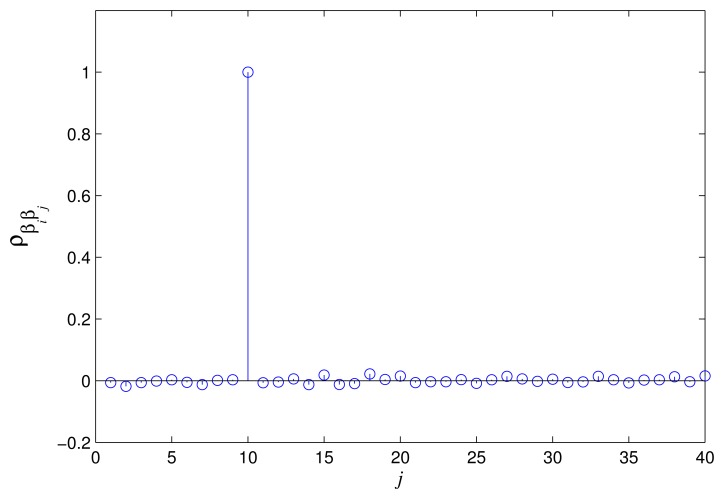
The correlation coefficient *ρ_βiβj_* when *i* = 10.

In Figures 3 and 4, the correlation coefficients of *α_i_*, *α_j_* and *β_i_*, *β_j_* are shown. We can see that both of the correlation coefficients (*ρ_αiαj_* and *ρ_βiβj_*) are nearly to zero when *i* ≠ *j*. Thus, *α_i_*, *α_j_* and *β_i_*, *β_j_* can be approximated as uncorrelated random variables, respectively. In order to get the specific expression for the distribution of the ${T^{\prime }}_{DC-CP}$, we assume that both the *α_i_*, *α_j_* and *β_i_*, *β_j_* are independent when *i* ≠ *j*. Note that although not theoretically correct, simulations have shown that this assumption has indeed very little effect on the true result and greatly simplified the derivation process. Therefore, the test statistic of DC-CP detector can be rewritten as

(58) $${T^{\prime \prime }}_{DC-CP}=\sum_{i=0}^{N_{c}+N_{d}-1}\frac{\alpha_{i}^{2}+\beta_{i}^{2}}{\sigma_{i}^{2}/2}\overset{H_{0}}{\underset{H_{1}}{≶}}{\gamma^{\prime \prime }}_{DC-CP}$$

where ${T^{\prime \prime }}_{DC-CP}$ obeys noncentral chi-square distribution that the degree of freedom is 2(*N_c_* + *N_d_*) and the non-centrality parameter is $\lambda_{DC-CP}=\sum_{i=0}^{N_{c}+N_{d}-1}\lambda_{\alpha_{i}}+\lambda_{\beta_{i}}$. Thus, under *H*_0_ and *H*_1_, the λ*_DC_*_−_*_CP_* can be written as

(59) $$\begin{array}{ll} \lambda_{DC-CP,0} & =\frac{M\sum_{i=0}^{N_{C}+N_{d}-1}1_{C}(i-\tau )}{2}\\ \lambda_{DC-CP,1} & =\frac{\sum_{i=0}^{N_{C}+N_{d}-1}2M1_{C}^{2}(i-\tau )\left(\theta^{2}+\sigma_{n}^{2}/2\right)}{\theta^{4}1_{C}(i-\tau )+{\left(\theta^{2}+\sigma_{n}^{2}\right)}^{2}} \end{array}$$

Therefore, the *P_f_, P_m_* and the threshold of DC-CP algorithm can be computed as

(60) $$\begin{array}{l} P_{f}=1-F\left({\gamma^{\prime \prime }}_{DC-CP};2,\lambda_{DC-CP,0}\right)\\ P_{m}=F\left({\gamma^{\prime \prime }}_{DC-CP};2,\lambda_{DC-CP,1}\right)\\ {\gamma^{\prime \prime }}_{DC-CP}=F^{-1}(1-P_{f};2,\lambda_{DC-CP,0}) \end{array}$$

Based on the conclusions mentioned above, the main spectrum sensing processes of the three algorithms can be summarized as follows.

(1) Compute the real parts and image parts of *R_i_* and *G_t_* as stated in Equations (20) and (40) using the received samples, respectively.
(2) Compute the test statistics of different methods with the priori knowledge. For the DC-OFDM detector, the *T_DC_*_−_*_OFDM_* is calculated according to Equations (17)–(19) for different *τ*. For the DC-PT detector and DC-CP detector, it can be computed based on Equations (54) and (58), respectively.
(3) Compute the thresholds of the three algorithms for a given *P_f_*. Specifically, for different *τ*, *γ_DC_*_−_*_OFDM_* can be got by applying Equations (51)–(53). According to Equations (56) and (60), thresholds of DC-PT and DC-CP can be calculated.
(4) Compare the test statistics of the proposed algorithms with their corresponding thresholds. If the test statistic is larger than the threshold, the primary user is present. Otherwise, the primary user is absent.

### 5.4. Computational Complexity Analysis

In this part, we compare our proposed algorithms with the traditional CP detector and PT detector in terms of computational complexity. We use the number of complex multiplications to measure the detectors' complexity.

For DC-OFDM detector, when *τ* ∈ [0,*N*_c_], *O*(3*MN_c_* + *MN_d_* − 2*τ*) complexity multiplications are required. So, when *τ* = *N_c_*, the minimum number of complexity multiplications is *O*((3*M* − 2)*N_c_* + *MN_d_*); When *τ* ∈ [*N_c_*+1, *N_d_* − 1], according to Equation (18), DC-OFDM detector costs *O*((*3M*−2)*N_c_*+*MN_d_*) complexity multiplications; When *τ* ∈ [*N_d_, N_C_* + *N_d_*−1], as is shown in Equation (19), O((3*M* − 2)*N_c_*+ *MN_d_* + 2(*τ* − *N_d_*)) times of complexity multiplication are needed to sense the spectrum. Thus, if *τ* = *N_d_*, the complexity multiplications can be minimized to *O*((3*M* − 2)*N_c_* + *MN_d_*). Based on the analysis above, the minimal computational complexity of DC-OFDM detector is *O*((3*M* − 2)*N_c_*+ *MN_d_*).

Moreover, according to Equations (56) and (58), the computational complexity of the DC-CP and DC-PT detectors are mainly caused by computing *R_i_* and *G_t_*. Thus, computational complexity for the DC-CP based method and DC-PT based method are *O*(*M*(*N_c_*+*N_d_*)) and *O*(*MN_d_*log(*N_d_*)+*M^2^N_p_/*2*t^2^*), respectively. Furthermore, the CP detector in [19] and PT detector in [21] require *O*(*M*(*N_c_* + *N_d_*)) and *O*(*MN_d_*log(*N_d_*) + *M^2^N_p_*/2*t^2^)* complexity multiplications. Note that DC-CP detector and DC-PT detector do not cost additional complexity multiplications compared with original CP detector and PT detector. Table 1 shows the computational complexity of different algorithms.

## 6. Simulation Results

In this section, some numerical results of the proposed schemes are given for sensing OFDM signals over frequency selective fading channel, and the performance of the algorithms is indicated as the probability of missed-detection (*P_m_*) via 10^6^ Monte Carlo simulations. During the simulation processes, we fix the probability of false alarm *P_f_* = 0.05 to get the thresholds and *P_m_*. The OFDM block size is chosen as *N_d_* = 32 and the CP is set to *N_c_* = *N_d_*/4, the PT length is *N_p_* = 10. The total number of the received OFDM blocks is *M* = 30. Moreover, the performance of these presented detectors with different parameters could also be shown.

**Figure 5:**
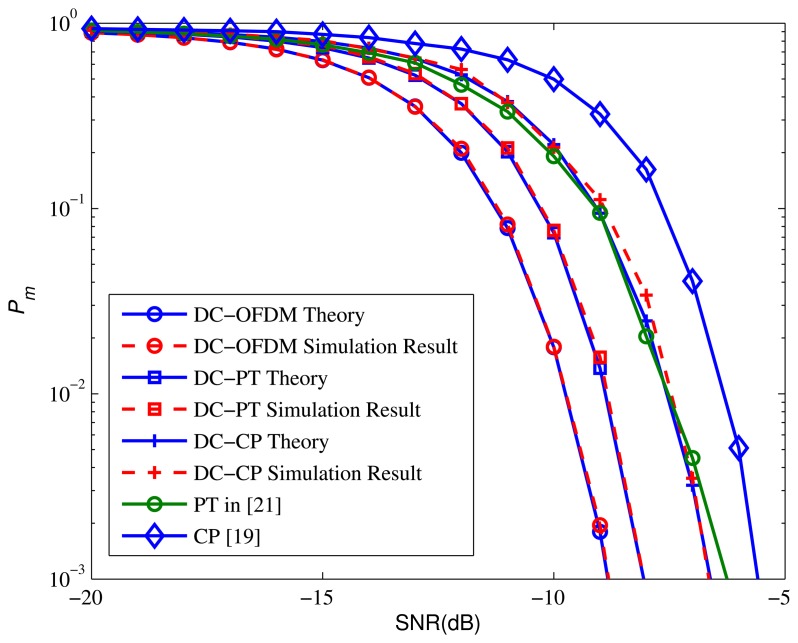
*P*_m_ performance comparison of proposed methods against other methods.

Figure 5 compares the performance of proposed algorithms with the algorithms in [19,21]. We assume *τ* = 15, so we take Equation (44) as the test statistic of DC-OFDM detector. It is apparent that the DC-OFDM detector achieves significant performance that it outperforms the other detectors. More specifically, the *P_m_* of DC-OFDM detector is just equal to 0.00195 when SNR = −9, while the *P_m_* of other detectors are quite high. On the other hand, the results show that both the DC-CP and DC-PT algorithms outperform the original methods by utilizing the differential operation to get the locally optimal solution to detect the signals.

**Figure 6:**
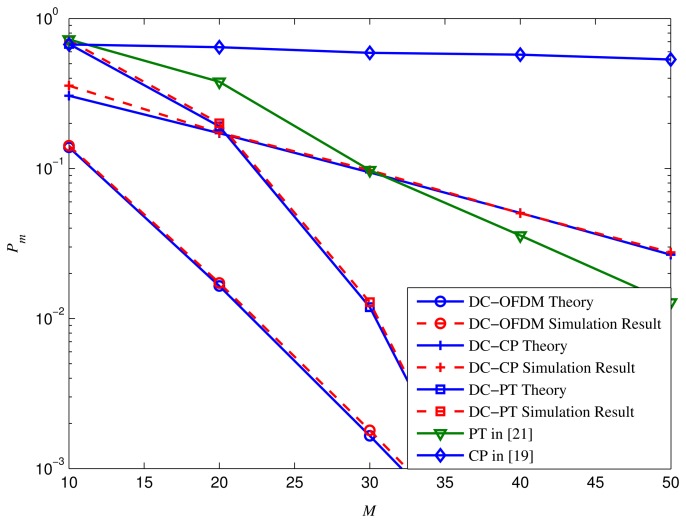
*P_m_* performance of different detectors with different numbers (*M*) of received OFDM blocks under SNR = −12 dB.

Figure 6 is the performance comparison between different detectors with different numbers of the OFDM blocks (which is denoted as *M*), where *N_d_* = 32,*N_c_* = *N_d_*/4, *N_p_* = 10, SNR = −9 dB and *P_f_* = 0.05. It is clear that the *P_m_* of CP detector and DC-CP detector decrease slightly by growing *M*, whereas it reduces faster for DC-OFDM detector, PT detector and DC-PT detector, respectively. Since large *M* costs longer sensing time, it is better to select a proper number of *M* to achieve a satisfied tradeoff between the sensing performance and detection time.

**Figure 7:**
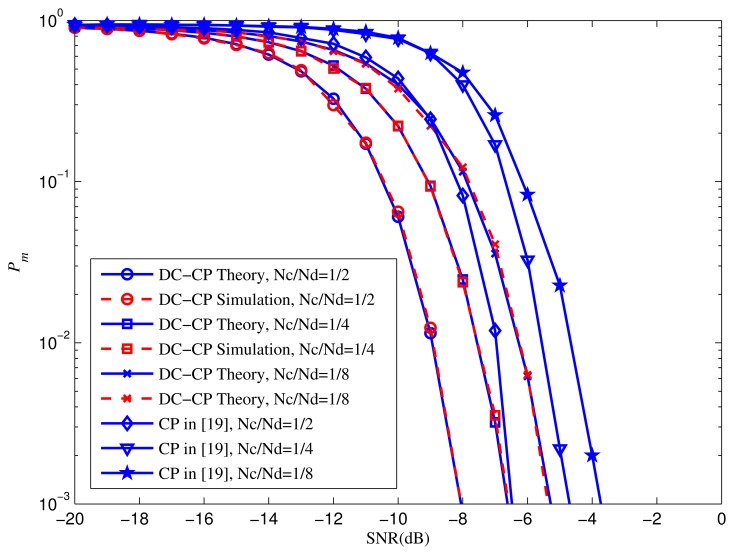
*P_m_* performance of DC-CP detection against traditional CP detection with *N_c_*/*N_d_* = 1/2, 1/4, 1/8.

The impact of the CP ratio is illustrated in Figure 7. In order to get the effect of CP ratio, the *N_c_* are set as *N_c_* = 1/2*N_d_*, 1/4*N_d_*, and 1/8*N_d_*, respectively. *N_d_* = 32, *M* = 10 and *P_f_* = 0.05. As is shown, the *P_m_* of both approaches decrease when the CP radio rises. Notably, the performance of DC-CP detector with different CP ratios expands faster than CP detector. In other words, with higher CP ratio, the DC-CP could achieve better outcome. In other words, our method can make better utilize of CP.

**Figure 8:**
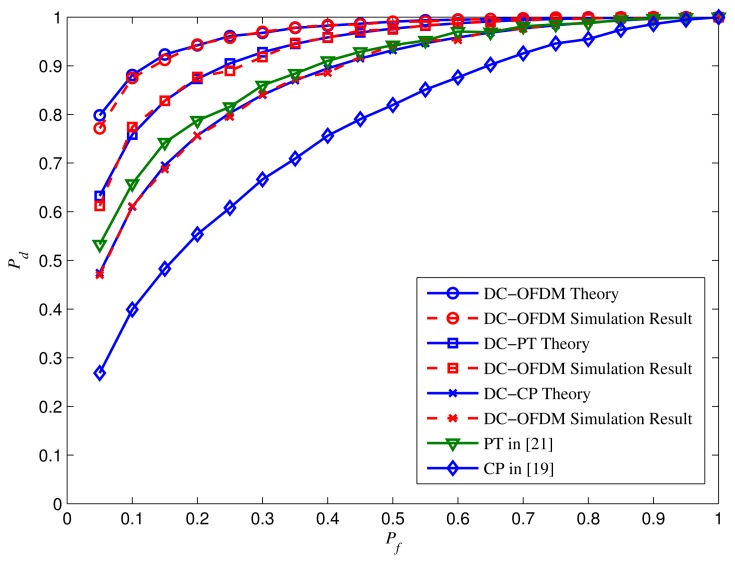
ROC curves for different algorithms at SNR = −12 dB.

The ROC curves for different algorithms at SNR = −12 dB are shown in Figure 8 by taking the same parameters in Figure 5. Simulation results show that the probability of detection *P_d_* of DC-OFDM detector approaches to 1 when *P_f_* is small, while other algorithms can achieve satisfied *P_d_* only when *P_f_* is relatively higher. Besides, the proposed approaches have the better performance compared with the traditional ones.

Noise uncertainty is an important factor which would affect the performance of the detector. In this paper, we assume that the accurate noise power $\sigma_{n}^{2}$ is obtained, while it is hard to be achieved in the practical application. Thus, it is necessary to discuss the influence of noise uncertainty for our proposed detectors.

**Figure 9:**
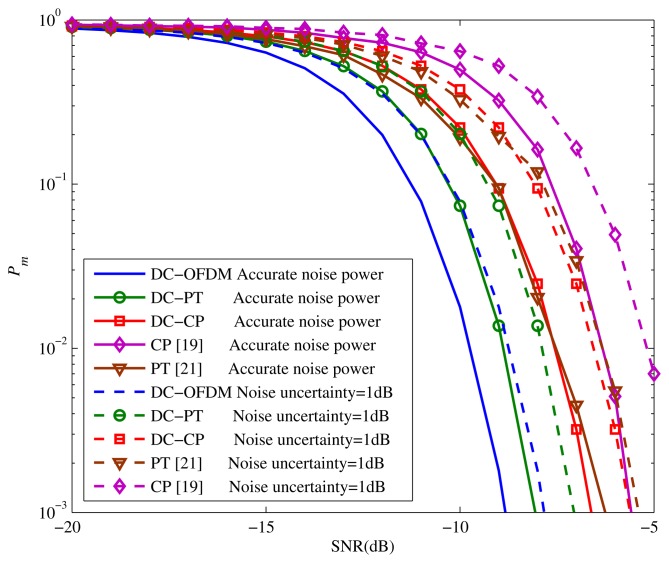
*P_m_* performance of proposed detectors with noise uncertainty.

Figure 9 is the performance of different detectors with noise uncertainty. The *P_f_* is set to 0.05, and noise uncertainty is equal to 1dB. We can see that with the increase of noise uncertainty, all of the detectors' results degrade correspondingly. Although noise uncertainty would cause some performance degradations, *P_m_* of our proposed detectors are still in the satisfied range. Take the DC-PT detector as an example, when noise uncertainty is 1dB, it is still better than the original PT detector with no noise uncertainty. Moreover, when SNR = −9 dB, the *P_m_* of DC-OFDM detector with 1dB noise uncertainty is only 0.01783 which is quite smaller than other detectors. So when the noise uncertainty is small, the proposed detectors are effective to complete the spectrum sensing.

**Figure 10:**
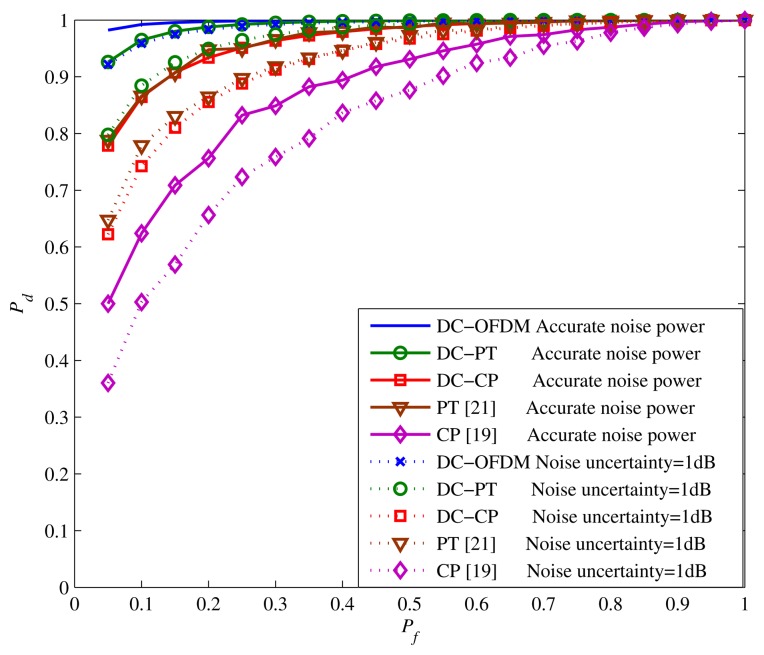
ROC curves for different detectors with different noise at SNR = −10 dB.

The similar conclusions can be derived according to the ROC curves with different noise uncertainty shown in Figure 10 when SNR = −10 dB. It is apparent that the noise uncertainty could impact the *P_d_* of all detectors. However, the decreasing of *P_d_* is not huge when noise uncertainty is small, and all of the proposed detectors can achieve the good detection performance. For instance, when noise uncertainty is 1 dB, the DC-CP detector is markedly better than the CP detector with no noise uncertainty. Therefore, based on Figures 9 and 10 we can make the conclusion that although the proposed detectors do not have a very strong ability to resist noise uncertainty, they could still get the novel sensing performance when the accurate or relatively accurate noise power could be obtained.

**Figure 11:**
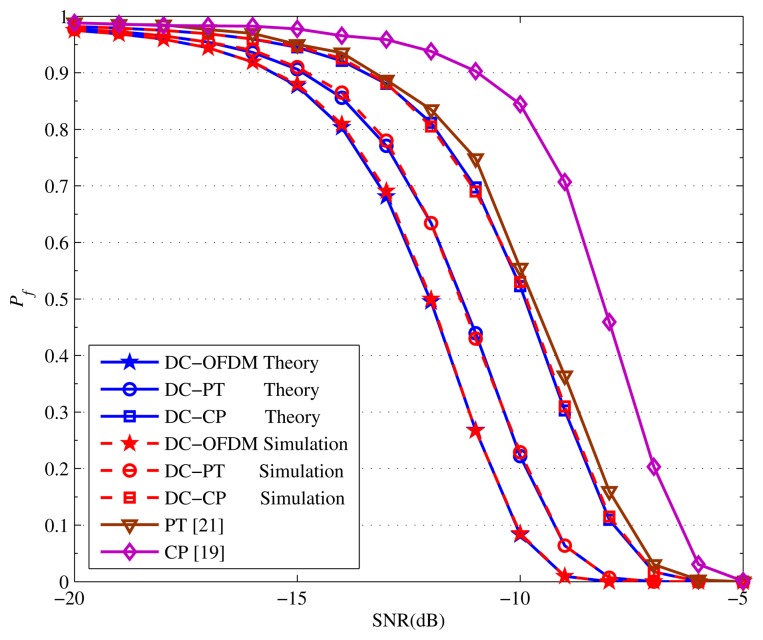
*P*_f_ performance of different detectors when *P_d_* = 0.99.

**Figure 12:**
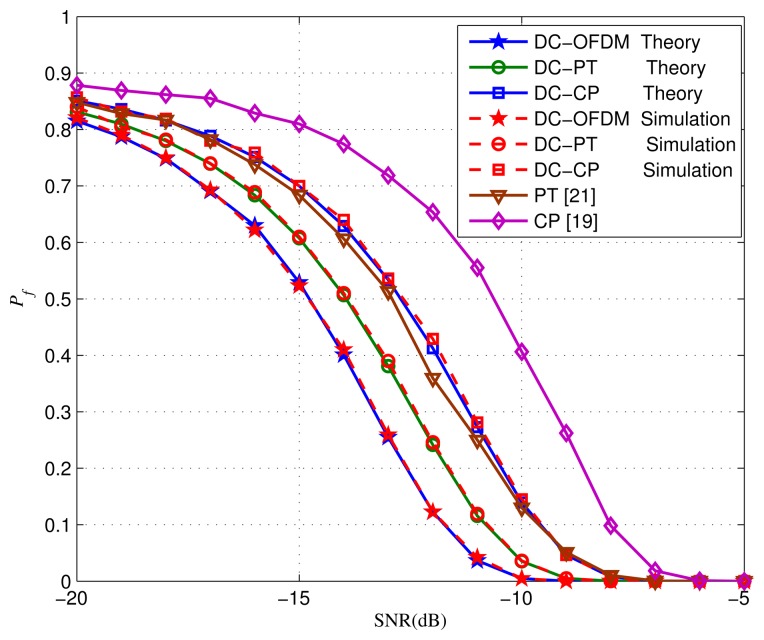
*P_f_* performance of different detectors when *P_d_* = 0.9.

Finally, the *P_f_* performance when *P_d_* = 0.99 and *P_d_* = 0.9 are indicated in Figures 11 and 12, respectively. Obviously, the DC-OFDM detector has the lowest *P_f_* to obtain a required *P_d_* among the five detectors. Moreover, when *P_d_* is fixed, with the SNR gradually improving, the *P_f_* of all the detectors decrease dramatically. Remarkably our proposed detectors are always smaller than the corresponding original detectors. For example, as is shown in Figure 12, when *P_d_* = 0.9 and SNR = −15 dB, the *P_f_* of DC-PT detector is just 0.6075, but the PT detector's is up to 0.692. Therefore, considering the *P_f_* performance, our proposed detectors have great advantages compared with other detectors.

## 7. Conclusions

In this paper, three new spectrum sensing algorithms for OFDM signals are investigated under low SNR environment with the presence of a timing delay. We have proposed a DC-OFDM detection algorithm based on the differential characteristics for sensing the OFDM signals with the knowledge of noise power. The numerical comparisons show that the DC-OFDM detector can achieve the best detection performance among all the detectors considered in this paper. In addition, it provides a new way to improve the existing sensing approaches. The DC-CP detector and the DC-PT detector are two typical examples of taking differential operation to enhance the system performance. In particular, the DC-CP detector is based on the second order statistics of the OFDM signals, and the DC-PT detector is based on PT through frequency-domain cross-correlation. The simulation results indicate that the DC-CP detector outperforms the traditional CP detector, and it can make better utilize of the CP. Moreover, the DC-PT detector achieves a great improvement compared with PT detector. In this paper, we just analyze two traditional OFDM detectors (CP detector and PT detector), while there are many other detectors of sensing the OFDM signals that can employ differential operation to improve the system performance. This is a topic for our future research.

## Figures and Tables

**Table 1:** Computational complexity comparison.

**Method**	**Computational Complexity**
DC-OFDM detector	*O*((3*M* −2)*N_c_* +*MN_d_*)
CP detector	*O*(*M*(*N_c_* + *N_d_*))
DC-CP detector	*O*(*M*(*N_c_* + *N_d_*))
PT detector	*O*(*MN_d_*log(*N_d_*) + *M^2^N_p_*/2*t*^2^)
DC-PT detector	*O*(*MN_d_*log(*N_d_*) + *M^2^N_p_*/2*t*^2^)

## Appendix

### A. The Expression of $A_{1}^{\left(1\right)}$

According to the expression of *A*_1_, the first derivative of it can be shown as:

(A1) $$\begin{array}{ll} A_{1}^{\left(1\right)}= & -\frac{(M-1)(N_{d}-N_{c})+2\tau }{2}\left(1+\frac{\theta^{2}}{\sigma_{n}^{2}}\right)-\frac{(M-1)(N_{d}-N_{c})+2\tau }{2}-1\left(\frac{2\theta }{\sigma_{n}^{2}}\right)\left(1+\frac{2\theta^{2}}{\sigma_{n}^{2}}\right)-\frac{(M-1)N_{c}-\tau }{2}\\ & -\frac{(M-1)N_{c}-\tau }{2}\left(1+\frac{2\theta^{2}}{\sigma_{n}^{2}}\right)-\frac{(M-1)N_{c}-\tau }{2}-1\left(\frac{4\theta }{\sigma_{n}^{2}}\right)\left(1+\frac{\theta^{2}}{\sigma_{n}^{2}}\right)-\frac{(M-1)(N_{d}-N_{c})+2\tau }{2} \end{array}$$

### B. The Expression of $A_{2}^{\left(1\right)}$

Based on the definition of *A*_2_, the first derivative of *A*_2_ can be written as:

(B1) $$\begin{array}{ll} A_{2}^{\left(1\right)} & =\sum_{m=0}^{M-1}\sum_{i=0}^{N_{c}-\tau -1}x_{m}(i)*\left(\left(\frac{2\theta }{2\theta^{2}\sigma_{n}^{2}+\sigma_{n}^{4}}-\frac{\left(4\theta \sigma_{n}^{2})(\theta^{2}+\sigma_{n}^{2}\right)}{{\left(2\theta^{2}\sigma_{n}^{2}+\sigma_{n}^{4}\right)}^{2}}\right)x_{m}(i)-\left(\frac{2\theta }{2\theta^{2}\sigma_{n}^{2}+\sigma_{n}^{4}}-\frac{\left(4\theta \sigma_{n}^{2})(\theta^{2}\right)}{{\left(2\theta^{2}\sigma_{n}^{2}+\sigma_{n}^{4}\right)}^{2}}\right)x_{m}(i+N_{d})\right)\\ & +\sum_{i=N_{d}}^{N_{c}+N_{d}-\tau -1}x_{0}(i)*\left(\left(\frac{2\theta }{2\theta^{2}\sigma_{n}^{2}+\sigma_{n}^{4}}-\frac{\left(4\theta \sigma_{n}^{2})(\theta^{2}+\sigma_{n}^{2}\right)}{{\left(2\theta^{2}\sigma_{n}^{2}+\sigma_{n}^{4}\right)}^{2}}\right)x_{0}(i)-\left(\frac{2\theta }{2\theta^{2}\sigma_{n}^{2}+\sigma_{n}^{4}}-\frac{\left(4\theta \sigma_{n}^{2})(\theta^{2}\right)}{{\left(2\theta^{2}\sigma_{n}^{2}+\sigma_{n}^{4}\right)}^{2}}\right)x_{0}(i-N_{d})\right)\\ & +\sum_{m=0}^{M-2}\sum_{i=N_{c}+N_{d}-\tau }^{N_{c}+N_{d}-1}x_{m}(i)*\left(\left(\frac{2\theta }{2\theta^{2}\sigma_{n}^{2}+\sigma_{n}^{4}}-\frac{\left(4\theta \sigma_{n}^{2})(\theta^{2}+\sigma_{n}^{2}\right)}{{\left(2\theta^{2}\sigma_{n}^{2}+\sigma_{n}^{4}\right)}^{2}}\right)x_{m}(i)-\left(\frac{2\theta }{2\theta^{2}\sigma_{n}^{2}+\sigma_{n}^{4}}-\frac{\left(4\theta \sigma_{n}^{2})(\theta^{2}\right)}{{\left(2\theta^{2}\sigma_{n}^{2}+\sigma_{n}^{4}\right)}^{2}}\right)x_{m}(i+N_{d})\right)\\ & +\sum_{m=1}^{M-1}\sum_{i=N_{d}-\tau }^{N_{c}+N_{d}-\tau -1}x_{m}(i)*\left(\left(\frac{2\theta }{2\theta^{2}\sigma_{n}^{2}+\sigma_{n}^{4}}-\frac{\left(4\theta \sigma_{n}^{2})(\theta^{2}+\sigma_{n}^{2}\right)}{{\left(2\theta^{2}\sigma_{n}^{2}+\sigma_{n}^{4}\right)}^{2}}\right)x_{m}(i)-\left(\frac{2\theta }{2\theta^{2}\sigma_{n}^{2}+\sigma_{n}^{4}}-\frac{\left(4\theta \sigma_{n}^{2})(\theta^{2}\right)}{{\left(2\theta^{2}\sigma_{n}^{2}+\sigma_{n}^{4}\right)}^{2}}\right)x_{m}(i-N_{d})\right)\\ & +\sum_{i=N_{c}-\tau }^{N_{d}-\tau -1}{\left|x_{m}(i)\right|}^{2}\left(-\frac{2\theta }{{\left(\theta^{2}+\sigma_{n}^{2}\right)}^{2}}\right)]+\sum_{i=N_{c}-\tau }^{N_{d}-1}{\left|x_{0}(i)\right|}^{2}\left(-\frac{2\theta }{{\left(\theta^{2}+\sigma_{n}^{2}\right)}^{2}}\right)+\sum_{i=N_{c}+N_{d}-\tau }^{N_{c}+N_{d}-1}{\left|x_{M-1}(i)\right|}^{2}\left(-\frac{2\theta }{{\left(\theta^{2}+\sigma_{n}^{2}\right)}^{2}}\right) \end{array}$$

### C. The Expression of $D_{1}^{\left(1\right)}$

Equation (30) is the expression of *D*_1_, thus $D_{1}^{\left(1\right)}$ can be expressed as:

(C1) $$D_{1}^{\left(1\right)}=-\frac{1}{2}\frac{4\theta^{3}1_{C}^{2}(i-\tau )M+2M(\theta^{2}+\sigma_{n}^{2})(2\theta )}{{\left(\theta^{4}1_{C}^{2}(i-\tau )M+M{\left(\theta^{2}+\sigma_{n}^{2}\right)}^{2}\right)}^{\frac{3}{2}}}$$

### D. The Expression of $D_{2}^{\left(1\right)}$

(D1) D2(1)=−12(−4Re(Ri)Mθ1C(i−τ)+4M2θ31C2(i−τ)θ41C2(i−τ)M+M(θ2+σn2)2−(4Mθ31C2(i−τ)+2M(θ2+σn2)2θ)|Ri−θ2M1C(i−τ)|2(θ4textbf1C2(i−τ)M+M(θ2+σn2)2)2

### E. Appendix: The Expressions of $D_{1}^{\left(2\right)}$ and $D_{2}^{\left(2\right)}$

According to Equations (C1) and (D1), the second derivative of *D*_1_ and *D*_2_ are

(E1) $$\begin{array}{ll} D_{1}^{\left(2\right)} & =-\frac{1}{2}[-\frac{3}{2}\frac{{\left(4\theta^{3}1_{C}^{2}(i-\tau )M+2M(\theta^{2}+\sigma_{n}^{2})(2\theta )\right)}^{2}}{{\left(\theta^{3}1_{C}^{2}(i-\tau )M+M{\left(\theta^{2}+\sigma_{n}^{2}\right)}^{2}\right)}^{\frac{5}{2}}}\\ & +\frac{12\theta^{2}1_{C}^{2}(i-\tau )M+(2\theta )(4\theta M)+4M(\theta^{2}+\sigma_{n}^{2})}{{\left(\theta^{4}1_{C}^{2}(i-\tau )M+M{\left(\theta^{2}+\sigma_{n}^{2}\right)}^{2}\right)}^{\frac{3}{2}}}] \end{array}$$

(E2) D2(2)=−12[−4Re(Ri)M1C(i−τ)+12M2θ21C2(i−τ)θ41C2(i−τ)M+M(θ2+σn2)2−(4Mθ31C2(i−τ)+2M(θ2+σn2)(2θ))(θ41C2(i−τ)M+M(θ2+σn2)2)2×(−4Re(Ri)Mθ1C(i−τ)+4M2θ31C2(i−τ))−−2(4Mθ31C2(i−τ)+2M(θ2+σn2)(2θ))2|Ri−θ2M1C(i−τ)|2(θ41C2(i−τ)M+M(θ2+σn2)2)3+12Mθ31C2(i−τ)M+(2θ)(4θM)+4M(θ2+σn2)|Ri−θ2M1C(i−τ)|2(θ41C2(i−τ)M+M(θ2+σn2)2)2+(−4Re(Ri)Mθ1C(i−τ)+4M2θ31C2(i−τ))(θ41C2(i−τ)M+M(θ2+σn2)2)2×(4Mθ31C2(i−τ)+2M(θ2+σn2)(2θ))]

### F. The Means, Variances and the Covariance of *I_i_*

According to the definition of *I*_1_, under *H*_0_, the received signal only contains noise. Thus the mean of *I*_1_ can be written as

(F1) $$\mu_{I_{1}}=\text{E}[\sum_{m=0}^{M-1}\sum_{i=0}^{N_{c}+N_{d}-1}{\left|e_{m}(i)\right|}^{2}]=M(N_{c}+N_{d})\sigma_{n}^{2}$$

For the variance of *I*_1_, it can be computed as

(F2) $$\sigma_{I_{1}}^{2}=\text{E}[{\left|\sum_{m=0}^{M-1}\sum_{i=0}^{N_{c}+N_{d}-1}{\left|e_{m}(i)\right|}^{2}\right|}^{2}]-{\left|\mu_{I_{1}}\right|}^{2}$$

Let $W_{m}=\sum_{i=0}^{N_{c}+N_{d}-1}{\left|e_{m}\left(i\right)\right|}^{2}$, then $\sigma_{I_{1}}^{2}=\text{E}\left[{\left|\sum_{m=0}^{M-1}W_{m}\right|}^{2}\right]-{\left|\mu_{I_{1}}\right|}^{2}$. Now, we compute $\text{E}\left[{\left|\sum_{m=0}^{M-1}W_{m}\right|}^{2}\right]$ that

(F3) $$\begin{array}{ll} \text{E}[{\left|\sum_{m=0}^{M-1}W_{m}\right|}^{2}] & =\text{E}[\sum_{m=0}^{M-1}W_{m}^{2}+2\overset{M-1}{\overset{︷}{(W_{0}^{*}W_{1}+W_{0}^{*}W_{2}+\cdots +W_{0}^{*}W_{M-1}}}\\ & +\overset{M-2}{\overset{︷}{W_{1}^{*}W_{2}+W_{1}^{*}W_{3}+\cdots +W_{1}^{*}W_{M-1}}}+\cdots +W_{M-2}^{*}W_{M-1})]\\ & =\sum_{m=0}^{M-1}{\text{E}[\text{W}}_{m}^{2}]+\frac{M-1}{M}{\left(\text{E}[\sum_{m=0}^{M-1}W_{m}]\right)}^{2}\\ & =\sum_{m=0}^{M-1}{\text{E}[\text{W}}_{m}^{2}]+\frac{M-1}{M}\mu_{I_{1}}^{2} \end{array}$$

As is shown in Equation (F3), it contains two parts. For the first part, it is equal to

(F4) $$\begin{array}{l} \sum_{m=0}^{M-1}{\text{E}[\text{W}}_{m}^{2}]=\sum_{m=0}^{M-1}\text{E}[{\left(\sum_{i=0}^{N_{c}+N_{d}-1}{\left|e_{m}(i)\right|}^{2}\right)}^{2}]\\ M\text{E}[\sum_{i=0}^{N_{c}+N_{d}-1}{\left|e_{m}(i)\right|}^{4}+2\overset{N_{c}+N_{d}-1}{\overset{︷}{\left(e_{m}(0)*e_{m}(1)+e_{m}(0)*e_{m}(2)+\cdots +e_{m}(0)*e_{m}(N_{c}+N_{d}-1\right)}}\\ +\overset{M-2}{\overset{︷}{e_{m}(1)*e_{m}(2)+e_{m}(1)*e_{m}(3)+\cdots +e_{m}(1)*e_{m}(N_{c}+N_{d}-1)}}+\ldots \\ +\overset{1}{\overset{︷}{e_{m}(N_{c}+N_{d}-2)*e_{m}(N_{c}+N_{d}-1))}}]\\ =M(N_{c}+N_{d})(N_{c}+N_{d}+1)\sigma_{n}^{4} \end{array}$$

Thus, the variance of *I*_1_ under *H*_0_ is

(F5) $$\sigma_{I_{1}}^{2}=M(N_{c}+N_{d})\sigma_{n}^{4}$$

Under *H*_1_, the mean of *I*_1_ is

(F6) $$\begin{array}{ll} \mu_{I_{1}} & =\text{E}[\sum_{\text{m}=0}^{M-1}\sum_{\text{i}=0}^{N_{c}+N_{d}-1}{\left|{\text{hs}}_{\text{m}}(\text{i}-\tau )+{\text{e}}_{m}(\text{i})\right|}^{2}]\\ & =M(N_{c}+N_{d})\text{E}[{\left|hs_{m}(i)\right|}^{2}+(hs_{m}(i))*{\text{e}}_{m}(i)+e_{m}(i)*(hs_{m}(i))+{\left|e_{m}(i)\right|}^{2}]\\ & =M(N_{c}+N_{d})(\theta^{2}+\sigma_{n}^{2}) \end{array}$$

and the variance of *I*_1_ is $\sigma_{I_{1}}^{2}=\text{E}\left[{\left|\sum_{m=0}^{M-1}\sum_{i=0}^{N_{c}+N_{d}-1}{\left|hs_{m}\left(i-\tau \right)e_{m}\left(i\right)\right|}^{2}\right|}^{2}\right]-{\left|\mu_{I_{1}}\right|}^{2}$. Employing the same derivation method discussed above, $\sigma_{I_{1}}^{2}$ is equal to

(F7) $$\sigma_{I_{1}}^{2}=M(N_{c}+N_{d}){\left(\theta^{2}+\sigma_{n}^{2}\right)}^{2}$$

Therefore, the mean and variance of *I*_1_ is

(F8) $$\{\begin{array}{lll} H_{0} & : & \mu_{I_{1}}=M\left(N_{c}+N_{d}\right)\sigma_{n}^{2}\\ & & \sigma_{I_{1}}^{2}=M\left(N_{c}+N_{d}\right)\sigma_{n}^{4}\\ H_{1} & : & \mu_{I_{1}}=M\left(N_{c}+N_{d}\right)\left(\theta^{2}+\sigma_{n}^{2}\right)\\ & & \sigma_{I_{1}}^{2}=M\left(N_{c}+N_{d}\right){\left(\theta^{2}+\sigma_{n}^{2}\right)}^{2} \end{array}$$

For the means and variances of *I*_2_ and *I*_3_, since the derivation processes are the same as *I*_1_, so we directly give the expressions of them.

(F9) $$\{\begin{array}{lll} H_{0} & : & \mu_{I_{2}}=0\\ & & \sigma_{I_{2}}^{2}=(M-1)N_{c}\sigma_{n}^{4}\\ H_{1} & : & \mu_{I_{2}}=(M-1)N_{c}\theta^{2}\\ & & \sigma_{I_{2}}^{2}=(M-1)N_{c}{\left(\theta^{2}+\sigma_{n}^{2}\right)}^{2} \end{array}$$

(F10) $$I_{3}\{\begin{array}{lll} H_{0} & : & \mu_{I_{3}}=0\\ & & \sigma_{I_{3}}^{2}=(M-1)N_{c}\sigma_{n}^{4}\\ H_{1} & : & \mu_{I_{3}}=(M-1)N_{c}\theta^{2}\\ & & \sigma_{I_{3}}^{2}=(M-1)N_{c}{\left(\theta^{2}+\sigma_{n}^{2}\right)}^{2} \end{array}$$

Next, the covariance of *I*_1_, *I*_2_ and *I*_3_ will be given. According to the definition of covariance, COV(*I*_1_*I*_2_) can be written as $\text{COV}\left(I_{1}I_{2}\right)=\text{E}\left[I_{1}I_{2}^{*}\right]-\text{E}\left[I_{1}\right]\text{E}\left[I_{2}\right]$. Here, E[*I*_1_] and E[*I*_2_] can be got by using Equations (F8) and (F9). And under *H*_0_, the $\text{E}\left[I_{1}I_{2}^{*}\right]$ can be computed as

(F11) $$\begin{array}{ll} \text{E}[{\text{I}}_{1}{\text{I}}_{2}^{*}] & =\text{E}[\left(\sum_{m=0}^{M-1}\sum_{i=0}^{N_{c}+N_{d}-1}{\left|e_{m}(i)\right|}^{2}\right)\left(\sum_{n=0}^{M-2}\sum_{j=N_{c}+N_{d}-\tau }^{2N_{c}+N_{d}-\tau -1}e_{n}^{*}(j)e_{n}(j+N_{d})\right)*]\\ & =M(M-1)\text{E}[\sum_{i=0}^{N_{c}+N_{d}-1}\sum_{j=N_{c}+N_{d}-\tau }^{2N_{c}+N_{d}-\tau -1}e_{m}^{*}(i)e_{m}(i)e_{n}(j)e_{n}^{*}(j+N_{d})]\\ & =0 \end{array}$$

As the result, COV(*I*_1_*I*_2_) = 0. It is easy to proof that COV(*I*_1_*I*_3_) = 0 and COV(*I*_2_*I*_3_) = 0 by using the same method.

On the other hand, under *H*_1_, the $\text{E}\left[I_{1}I_{2}^{*}\right]$ is

(F12) $$\begin{array}{l} \text{E}[{\text{I}}_{1}{\text{I}}_{2}^{*}]\\ =\text{E}[\left(\sum_{m=0}^{M-1}\sum_{i=0}^{N_{c}+N_{d}-1}{\left|hs_{m}(i)+e_{m}(i)\right|}^{2}\right)\\ \times \left(\sum_{n=0}^{M-2}\sum_{j=N_{c}+N_{d}-\tau }^{2N_{c}+N_{d}-\tau -1}\left(hs_{n}(j)+e_{n}(j))*(hs_{n}(j+N_{d})+e_{n}(j+N_{d})\right)\right)*] \end{array}$$

Let

$$\begin{array}{ll} W_{m} & =\sum_{i=0}^{N_{c}+N_{d}-1}{\left|hs_{m}(i)+e_{m}(i)\right|}^{2}\\ & =\sum_{i=0}^{N_{c}+N_{d}-1}\left({\left|hs_{m}(i)\right|}^{2}+(hs_{m}(i))*e_{m}(i)+hs_{m}(i)e_{m}(i)*+{\left|e_{m}(i)\right|}^{2}\right) \end{array}$$

and

$$Q_{n}=\sum_{j=N_{c}+N_{d}-\tau }^{2N_{c}+N_{d}-\tau -1}\left(hs_{n}(j)+e_{n}(j))*(hs_{n}(j+N_{d})+e_{n}(j+N_{d}\right)$$

Then Equation (F12) can be written as

(F13) $$\begin{array}{ll} \text{E}[{\text{I}}_{1}{\text{I}}_{2}^{*}] & =\text{E}[\left(\sum_{m=0}^{M-1}W_{m}\sum_{n=0}^{M-2}Q_{n}\right)\\ & =\sum_{m=0}^{M-1}\sum_{\begin{array}{c} n=0\\ n\neq m \end{array}}^{M-2}{\text{E}[\text{W}}_{m}{+\text{Q}}_{n}]+\sum_{M=0}^{M-2}{\text{E}[\text{W}}_{m}{\text{Q}}_{m}]\\ & ={\left(M-1\right)}^{2}\left(N_{c}+N_{d}\right)N_{c}\left(\theta^{2}+\sigma_{n}^{2}\right)\theta^{2}+\left(M-1\right)\left(N_{c}+N_{d}+2\right)N_{c}\left(\theta^{2}+\sigma_{n}^{2}\right)\theta^{2} \end{array}$$

Thus, using Equations (F8), (F9) and (F13), COV(*I*_1_*I*_2_) is equal to $2\left(M-1\right)N_{c}\left(\theta^{2}+\sigma_{n}^{2}\right)\theta^{2}$. In a similar way, we can get that $\text{COV}\left(I_{1}I_{3}\right)2\left(M-1\right)N_{c}\left(\theta^{2}+\sigma_{n}^{2}\right)\theta^{2}$ and COV(*I*_2_*I*_3_*)* = 0.
